# Unique case of granulomatous arteritis in a grey mouse lemur *(Microcebus murinus)* – first case description

**DOI:** 10.5194/pb-4-71-2017

**Published:** 2017-04-03

**Authors:** Nicole Cichon, Karen Lampe, Felix Bremmer, Tamara Becker, Kerstin Mätz-Rensing

**Affiliations:** 1 German Primate Center, Kellnerweg 4, 37077 Göttingen, Germany; 2 Georg-August-Universität, Universitätsmedizin Göttingen, Robert Kochstr. 40, 37075 Göttingen, Germany

## Abstract

Overall, diseases of the vascular system are rarely observed entities among nonhuman primates that are commonly associated with systemic infections, septicemia or bacteremia. Rhesus monkeys infected with simian immunodeficiency virus (SIV) may develop a chronic occlusive arteriopathy of unknown etiology in late stages of the disease. This SIV associated arteriopathy is the only well-known specific vascular entity described in nonhuman primates. We herein report a unique case of granulomatous arteritis in a grey mouse lemur affecting multiple organs, which is not comparable to other disease entities formerly described in nonhuman primates. The features of the entity most closely resemble disseminated visceral giant cell arteritis in humans. A concise description of the disease is given, and the differential diagnoses are discussed. An idiopathic pathogenesis is suspected.

## 1 Introduction

**Figure 1:**
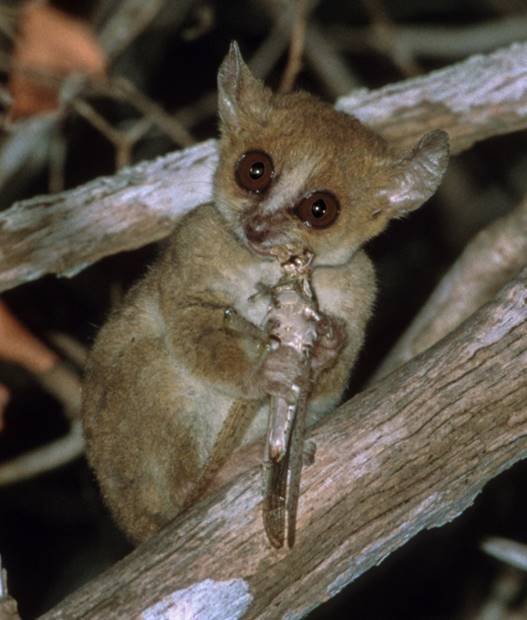
Grey mouse lemur (picture by Dr. Manfred Eberle).

Grey mouse lemurs (*Microcebus murinus) *are primates belonging to the suborder Strepsirrhini and infraorder Lemuriformes (Fig. 1). Within the Lemuriformes they belong to the family Cheirogaleidae, a group that includes the smallest primates in the world. Among this family the grey mouse lemur is a small-bodied lemur with an average weight of 60 g. Like all other members of the family Cheirogaleidae, grey mouse lemurs are nocturnal and arboreal. Mouse lemurs are important animal models in biomedical and basic biological research. They are used as an animal model for cerebral aging and neurodegenerative diseases (Fischer and Ausstad, 2011; Verdier et al., 2015). Their high species diversity makes them interesting and important for evolutionary research (Zimmermann and Radespiel, 2014). Meanwhile, their genome is completely sequenced by the Broad Institute (GenBank accession number ABDC00000000). Mouse lemurs in captivity suffer most frequently from chronic nephritis and renal insufficiency. This syndrome is accompanied by hormone imbalances characterized by increased cortico- and medulloadrenal secretion most probably induced by stress factors occurring in captivity (Perret, 1982). Vasculitis of any type has not been reported in mouse lemurs. We describe here a unique case of granulomatous vasculitis, which shares several features with an extremely rare entity in humans called disseminated visceral giant cell arteritis (DVGCA). DVGCA is an unusual type of extracranial giant cell arteritis involving arteries and arterioles of various organs with unknown pathogenesis. The entity was first described in 1978 by Lie and is only reported in humans. The type of vessels which are principally involved, the presence or absence of giant cells, vascular fibrinoid necrosis and eosinophilic infiltrates may help to distinguish the different vasculitis types (Lie, 1978; Jennette et al., 2013). The differences between the reported disease and other forms of vasculitis are discussed in detail.

## 2 Case report

Within a captive, indoor-housed colony of grey mouse lemurs, a 6-year-old intact male animal presented with acute onset of clinical symptoms including hematuria, reduced general condition, weight loss and inappetence. Physical examination revealed a poor body condition and a blood-smeared coat in the genital region. Injuries were not detected; thus, an acute hemorrhagic cystitis was suspected. Therapy consisted of parenteral application of enrofloxacin, meloxicam, fluid therapy, and vitamins and supplementary food as supportive care. Two days after initial presentation, the lemur was found dead.

## 3 Gross pathology and histology

At necropsy, hemorrhages were found within several organs. They were most prominent within lung parenchyma and urinary bladder and less severe within the renal pelvis and the subcutis. Furthermore, the spleen was enlarged.

**Figure 2:**
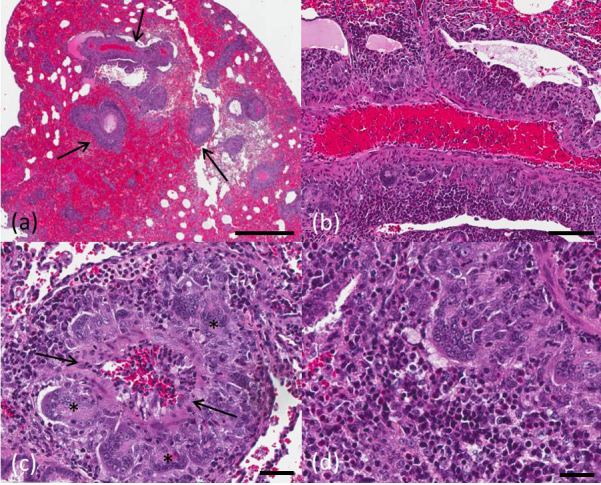
Grey mouse lemur, lung. **(a)** Perivascularly accentuated distribution pattern of the inflammation (arrows) and severe alveolar hemorrhage, scale bar 1 mm. **(b)** Granulomatous inflammation around a blood vessel, scale bar 100 µm. **(c)** Discreet fibrinoid necrosis of a vessel wall (arrow) accompanied by a severe inflammatory reaction with characteristic giant cells (asterisk), scale bar 40 µm. **(d)** Mixed inflammatory cell infiltrate with eosinophils in the vascular periphery, scale bar 30 µm. Hematoxylin and eosin stain.

The main histologic finding within the lung parenchyma was a severe granulomatous inflammation of small- and medium-sized arteries (Fig. 2a–d). The intima of affected vessels showed mild fibrinoid necrosis and proliferation. The tunica media and adventitia were heavily infiltrated by a mixed-cellular infiltrate. Giant cells of foreign body and Langhans type represented the dominant cell type. Eosinophils, a type of leucocytes, were also present in a moderate number. The lesions were accompanied by alveolar hemorrhage and histiocytosis. Vascular inflammation of milder degree was found within the kidneys and the liver. At these latter sites, giant cells were absent. Large foci of hemorrhages and perivascularly accentuated mixed-cellular inflammation were observed in the urinary bladder. Reactive extramedullary hematopoiesis was prominent within the spleen and, to a lesser degree, within the liver. Furthermore, a mild lymphocytic interstitial myocarditis was evident. The aorta was unremarkable.

**Table 1:** Relevant differential diagnoses for disseminated visceral giant cell arteritis.

Pathologic entity	Principle affected vessel	Giant cells	Fibrinoid necrosis	Eosinophilic infiltrates	ANCA
Giant cell arteritis, (GCA) (arteritistemporalis)	aorta, large systemicarteries	+	±	±	
Takayasu's arteritis (TAK)	aorta and aortic archbranches	±	–	–	
Polyarteritis nodosa (PAN)	medium-sized andsmall arteries	±	+++	+++	negative
Microscopic polyangiitis (MPA)	extracranial small arteries and veins	–	+++	±	positive
Eosinophilic granulo-matosis with polyangiitis (Churg–Straussvasculitis) (EGPA)	extracranial small arteries and veins, perivascular tissue	+	+++	+++	positive
Granulomatosis with polyangiitis (Wegenergranulomatosis) (GPA)	small vessel of upperrespiratory tract, lung,kidney	+++	+++	+++	positivec-ANCA
Disseminated visceral giant cell arteritis(DVGCA)	extracranial small arteries and arterioles	+++	±	±	negative

Legend: –: absent; ±: present occasionally; +: usually present; +++: always present (adapted to Lie, 1978, and Jennette et al., 2013).

In order to detect the cause of the granulomatous vasculitis and to reveal possible infectious pathogens like mycobacteria, fungi or even parasites, several histochemical stains were performed. Giemsa, Ziehl–Neelsen and Grocott staining as well as periodic acid-Schiff (PAS) reaction were negative. A routinely performed bacteriologic investigation of all organ systems revealed no pathogenic microorganisms within the tissues. Furthermore, lung tissue was tested by nested and real-time polymerase chain reaction (PCR) for the presence of a viral infection with influenza A or B, enterovirus, adenovirus, paramyxovirus, picornavirus and respiratory syncytial virus (RSV). There was no evidence of a viral or bacterial infection of the respiratory tract. Thus, the cause of disease remains unclear.

## 4 Discussion

A unique case of an idiopathic granulomatous generalized vasculitis in a mouse lemur is described. Inflammation of the blood vessel wall can be induced by several infectious agents in the course of bacteremia or septicemia. In the present case, no specific microorganisms were detectable by routine microbiological investigations. Furthermore, most important infectious diseases inducing granulomatous inflammation such as tuberculosis, leprosy, aspergillosis and leishmaniasis could be ruled out histologically by special stains and by bacteriologic investigation of tissue samples. No evidence existed of a foreign body reaction or foreign body disease by histologic investigation. A drug-induced vasculitis could be excluded as well because the animal never received drugs like propylthiouracil, methimazole, sulfasalazine, D-penicillamine or minocycline capable of inducing microscopic polyangiitis. Finally, the observed disease is not comparable with a well-known arteriopathy, a unique entity of vascular diseases occurring in immunosuppressed simian immunodeficiency virus (SIV)-infected rhesus monkeys, which is characterized by intimal and medial thickening and fibrosis occluding the vessel lumen (Chalifoux et al., 1992). Given that evidence of any form of immunosuppression is lacking in the mouse lemur, an idiopathic autoimmune or allergic disorder is suspected as the underlying disease in the present case.

Several different forms of idiopathic disseminated giant cell arteritis are well recognized in humans and should be discussed as a differential diagnosis for this case (Table 1).

They mainly differ in their distribution, and a rough classification can be done according to the vessel type involved. In this respect, giant cell arteritis (GCA) and Takayasu's arteritis (TAK) could be excluded, because they mainly affect the aorta and other large-sized vessels, which were not altered in the present case. With the same argumentation, polyarteritis nodosa (PAN), an idiopathic multisystemic necrotizing vasculitis, could also be excluded, because it mainly affects medium-sized vessels and spares lung vasculature. PAN is the only idiopathic vasculitis which has been reported in animals, including dogs, rats, mice, cats, pigs and a cynomolgus macaque (Porter et al., 2003). PAN predominantly affects medium-sized arteries (Jenette et al., 2013). The inflammation is most severe in the kidneys, gastrointestinal tract and heart. The lesions are often polyphasic and segmental within an artery and cause a grossly visible nodular thickening. The inflammatory infiltrates consist predominantly of macrophages and T-lymphocytes (Porter et al., 2003). In the present case, especially the lungs were affected, and there were no nodular lesions typical for PAN. The main differences to the present case exist in the lack of giant cells and the amount of fibrinoid necrosis. For the given reasons PAN was excluded as a possible diagnosis.

Another differential diagnosis is microscopic polyangiitis (MPA), a necrotizing vasculitis predominantly affecting small vessels (arterioles, venules and capillaries). The disease was initially considered a microscopic form of PAN. Glomerulonephritis is a characteristic finding, and granulomatous inflammation is absent (Jenette et al., 2013). The main target organs are the kidneys and lungs, with lesions also observed in the skin, muscles, brain and digestive tract (Iida et al., 2016). The kidneys of this mouse lemur did not reveal glomerulonephritis, and the character of the inflammation was granulomatous. Therefore, this disease was excluded as a differential diagnosis.

The eosinophilic nature of the lesions described in the present case could indicate another form of idiopathic vasculitis called eosinophilic granulomatosis with polyangiitis (EGPA), formerly known as Churg–Strauss vasculitis. It often involves small- to medium-sized vessels of the respiratory tract and is associated with asthma and allergic rhinitis (Cottin et al., 1999; Groh et al., 2015). As emphasized in the name, the predominance of eosinophils is an essential feature (Jennette et al., 2013). A few eosinophils were detected in the case of the mouse lemur, but giant cells represent the predominant cell type. Furthermore, no symptoms of respiratory impairment were described before death. For these reasons, EGPA is excluded as differential diagnosis.

The described lesion also showed similarities to the entity of granulomatosis with polyangiitis (GPA), previously known as Wegener granulomatosis. GPA usually involves small- to medium-sized vessels of the upper and lower respiratory tract (Wojciechowska et al., 2016). Pauci-immune glomerulonephritis is a common feature (Jennette et al., 2013; Takeuchi et al., 2016), but this lesion was not seen in the present case. Like in the present case, hematuria is a frequent finding. The main histologic criteria are the presence of giant cells, which was a very prominent feature of the present case, and fibrinoid necrosis of vessel walls, which was less obvious. GPA is generally characterized by antineutrophil cytoplasm antibodies (ANCA) (Wojciechowska et al., 2016). Unfortunately, it was not possible to measure ANCA in the present case to definitely exclude this differential diagnosis.

The pattern and character of the lesions in this lemur were very similar to those described in human cases of DVGCA. Key features of DVGCA in humans are involvement of extracranial arteries and arterioles of several organs (heart, lungs, kidneys, liver, pancreas, stomach, trachea, and occasionally aorta and other large arteries). Histologic similarities are the presence of giant cells, a mixed inflammatory cell infiltrate with eosinophils and, to a lesser extent, fibrinoid necrosis of the vessel walls (Lie, 1978). The inflammatory cell infiltrates predominantly consist of histiocytes, lymphocytes and plasma cells. Microorganisms and foreign bodies are not found. By these characteristics, DVGCA can be distinguished from other necrotizing and granulomatous forms of vasculitis.

The cause of DVGCA is unknown. Hypothetically, it might be linked to bacterial or viral infection, autoimmune disease, hypersensitivity and genetic factors. A human case is reported that clinically responded to immunosuppressive therapy (Kagata et al., 1999; Alguacil-Garcia et al., 1995). To our knowledge, this is the first case of granulomatous vasculitis with striking features similar to disseminated visceral giant cell arteritis in a nonhuman primate.

## Data Availability

The original data on the postmortem, histological and microbiological examinations can be provided upon request.
